# RETRACTION: Effect of Diabetic Foot Ulcers and Other Risk Factors on the Prevalence of Lower Extremity Amputation: A Meta‐Analysis

**DOI:** 10.1111/iwj.70219

**Published:** 2025-02-11

## Abstract

**RETRACTION**: ZhangH. , HuangC. , BaiJ. , and WangJ. , “Effect of Diabetic Foot Ulcers and Other Risk Factors on the Prevalence of Lower Extremity Amputation: A Meta‐Analysis,” International Wound Journal 20, no. 8 (2023): 3035–3047, 10.1111/iwj.14179.37095728 PMC10502264

The above article, published online on 24 April 2023, in Wiley Online Library (http://onlinelibrary.wiley.com/), has been retracted by agreement between the journal Editor in Chief, Professor Keith Harding; and John Wiley & Sons Ltd. Following an investigation by the publisher, all parties have concluded that this article was accepted solely on the basis of a compromised peer review process. In addition, the investigation found unattributed textual overlap between this article and another article by different authors [1]. The editors have therefore decided to retract the article. The authors disagree with the retraction. The authors disagree with the retraction.

**Reference:**

[1] C. Lin, J. Liu, and H. Sun, “Risk Factors for Lower Extremity Amputation in Patients with Diabetic Foot Ulcers: A Meta‐Analysis,” PLOS ONE 15, no. 9 (2020): e0239236, 10.1371/journal.pone.0239236.32936828 PMC7494323

**RETRACTION**: H. Zhang , C. Huang , J. Bai , and J. Wang , “Effect of Diabetic Foot Ulcers and Other Risk Factors on the Prevalence of Lower Extremity Amputation: A Meta‐Analysis,” International Wound Journal 20, no. 8 (2023): 3035–3047, 10.1111/iwj.14179.37095728 PMC10502264

The above article, published online on 24 April 2023, in Wiley Online Library (http://onlinelibrary.wiley.com/), has been retracted by agreement between the journal Editor in Chief, Professor Keith Harding; and John Wiley & Sons Ltd. Following an investigation by the publisher, all parties have concluded that this article was accepted solely on the basis of a compromised peer review process. In addition, the investigation found unattributed textual overlap between this article and another article by different authors [1]. The editors have therefore decided to retract the article. The authors disagree with the retraction. The authors disagree with the retraction.

## Reference

[1] C. Lin, J. Liu, and H. Sun, “Risk Factors for Lower Extremity Amputation in Patients with Diabetic Foot Ulcers: A Meta‐Analysis,” PLOS ONE 15, no. 9 (2020): e0239236, 10.1371/journal.pone.0239236.32936828 PMC7494323

